# Cas9-directed immune tolerance in humans—a model to evaluate regulatory T cells in gene therapy?

**DOI:** 10.1038/s41434-021-00232-2

**Published:** 2021-02-11

**Authors:** Dimitrios Laurin Wagner, Lena Peter, Michael Schmueck-Henneresse

**Affiliations:** 1 Berlin Institute of Health (BIH)—Center for Regenerative Therapies (B-CRT), Charité—Universitätsmedizin Berlin, Berlin, Germany; 2 Berlin Center for Advanced Therapies (BeCAT), Charité—Universitätsmedizin Berlin, Berlin, Germany; 3 Institute of Transfusion Medicine, Charité—Universitätsmedizin Berlin, Berlin, Germany; 4 Einstein Center for Regenerative Therapies, Charité—Universitätsmedizin Berlin, 13353 Berlin, Germany

**Keywords:** Genetic engineering, Immunology, Diseases

## Abstract

The dichotomic nature of the adaptive immune response governs the outcome of clinical gene therapy. On the one hand, neutralizing antibodies and cytotoxic T cells can have a dramatic impact on the efficacy and safety of human gene therapies. On the other hand, regulatory T cells (Treg) can promote tolerance toward transgenes thereby enabling long-term benefits of in vivo gene therapy after a single administration. Pre-existing antibodies and T cell immunity has been a major obstacle for in vivo gene therapies with viral vectors. As CRISPR-Cas9 gene editing advances toward the clinics, the technology’s inherent immunogenicity must be addressed in order to guide clinical treatment decisions. This review summarizes the recent evidence on Cas9-specific immunity in humans—including early results from clinical trials—and discusses the risks for in vivo gene therapies. Finally, we focus on solutions and highlight the potential role of Cas9-specific Treg cells to promote immune tolerance. As a “beneficial alliance” beyond Cas9-immunity, antigen-specific Treg cells may serve as a living and targeted immunosuppressant to increase safety and efficacy of gene therapy.

## Tregs in gene therapy

The interaction between the immune system and gene therapeutic agents in vivo largely determines long-term benefit of the treatment intervention [1]. On the one hand, neutralizing antibodies can block gene delivery, and cytotoxic T cells can reduce efficacy as well as pose a significant safety risk. On the other hand, immune tolerance to transgenes and vector components are associated with long-term transgene expression and clinical success in some trials [2, 3].

**Fig. 1:**
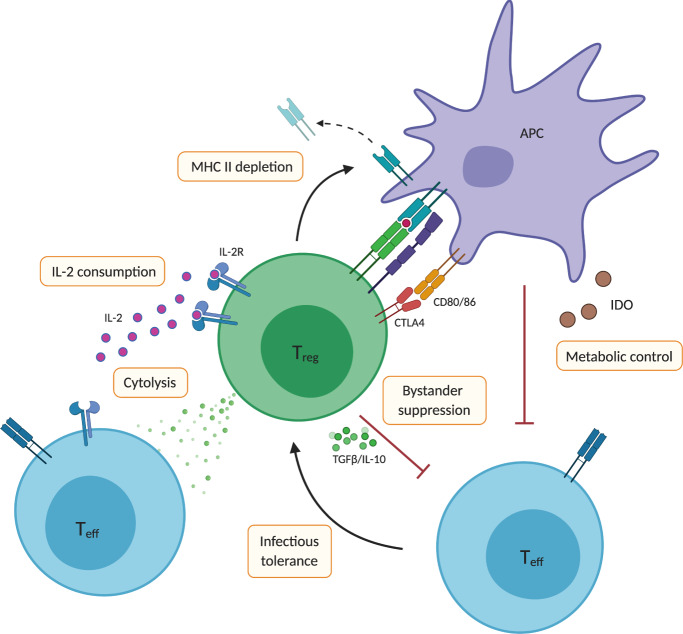
Suppressive actions of regulatory T cells. Regulatory T cells (Treg) are activated following recognition of specific antigens presented by antigen-presenting cells. However, Treg cells also exert a suppressive function irrespective of their antigen-specificity termed as bystander suppression. The mechanism of actions underlying the suppressive function of Tregs can be direct or indirect. Treg cells present with very high basal expression of high affinity CD25, enabling privileged consumption of the IL-2 growth factor and thereby depriving and weakening surrounding conventional effector T cells (Teff) cells. Further, Treg cells mediate specific suppression by depleting peptide-MHC class II-molecule complexes matching their TCR from the surface of antigen-presenting cells via trans-endocytosis and subsequent degradation. As opposed to boosting Teff cells by co-stimulatory signals, Treg cells carry molecules, which were classified as co-inhibitory receptors: e.g., CTLA-4 has high affinity to CD80/CD86 and thereby outcompetes co-stimulatory signals via CD28. Further, Treg cells can disrupt pro-inflammatory mechanisms by eliminating effector cells. Direct cell contact with Treg cells can also be fatal for the respective target cell by induction of the Fas-mediated pathway of apoptosis through ligation by Fas-ligand. In addition, Treg cells use perforin/granzyme dependent cytotoxicity to kill target cells accompanied by adhesion via CD18. Treg cells can also induce suppressive properties in adjacent cells by secreting soluble factors thereby inducing an *infectious tolerance*. For instance, human Treg cells secrete latent TGFβ thereby promoting a suppressive milieu, as well as shedding of soluble TNFα receptor II, which can neutralize TNFα and prevents its pro-inflammatory signaling. Notably, Tregs can also modulate their environment by secretion of IL-10. IL-2, interleukin 2; CD25, IL-2 receptor α chain; MHC, major histocompatibility complex; TCR, T cell receptor; CTLA-4, cytotoxic T-lymphocyte-associated Protein 4; CD80/CD86, B7, B7-2 type I membrane protein; CD28, co-stimulatory receptor for CD80/86; CD18, Integrin β-2; TGFβ, transforming growth factor; TNFα, tumor necrosis factor; IL-10, interleukin 10.

Among the mechanisms to induce and maintain immunological tolerance to antigens, diverse cell types play a central role, such as tolerogenic monocytes and dendritic cells as well as regulatory T cells (Tregs). Tregs can modulate cytotoxic and other effector T cell (Teff) immune responses, e.g., limiting of growth factors and thereby inhibiting their expansion or promoting dysfunctional Teff states (Brief summary in Fig. 1, reviewed extensively elsewhere [4, 5]). For example, in muscle-directed AAV therapy, Gernoux et al. showed that long-term transgene expression is associated with the simultaneous infiltration of Treg cells into the muscle and the exhaustion of Teff [6]. This is in line with previous results by Muller et al., who reported a similar phenomenon after muscular injection of AAV [3].

Treg cells are a multifunctional immunosuppressive cell type implicated in the control of overshooting immune responses and tissue regeneration. While thymus-derived Tregs cells are selected for self-antigen recognition, other T cells can be educated in the periphery to become antigen-specific Treg cells [7]. This has been described for commensal bacterial antigens and innocuous exogenous antigens at mucosal surfaces [8, 9].

A growing body of evidence highlights the importance of transgene- and vector-specific Treg cells for successful long-term benefits of in vivo gene therapy in animal models and humans (reviewed extensively elsewhere [6, 10]. In brief, multiple avenues have been explored for the specific induction of Treg cells to promote tolerance toward transgenes [10]: Delivery of viral vectors to the liver induces Treg cells and can be exploited to drive tolerance during co-delivery to other organs [11, 12]. Recently, this concept has been successfully applied in a mouse model of muscle gene therapy despite preexisting immunity directed against the transgene [13]. Alternatively, co-administration of AAV with tolerogenic rapamycin loaded nanoparticles has been shown to locally suppress immune responses in mouse studies, an effect which might have been amplified by a Treg-mediated effect [14]. Oral antigen uptake via modified plants has also been explored to induce antigen-specific Treg cells prior to vector application [15, 16].

As an alternative to Treg induction in vivo, infusion of ex vivo expanded polyclonal Treg cells has been shown to prevent detrimental immunity to viral vectors in mouse models [17]. Recently, another mouse study showed that in vitro expanded factor VIII-antigen-specific Tregs were superior to polyclonal Tregs in suppressing anti-factor VIII specific antibody formation after FVIII-overexpression with plasmids in mice [18]. To date, the role and potential of preexisting antigen-specific Treg cells for gene therapy has not been intensively studied in humans.

## CRISPR-Cas as a treatment modality to fix broken genes

The RNA-guided bacterial antiviral defense system CRISPR-Cas (clustered regularly interspaced short palindromic repeats–CRISPR-associated protein) repurposed for genome editing is becoming a powerful new tool to develop potent therapies for inherited diseases [19]. In contrast to conventional gene therapy that relies on overexpression of transgenes, CRISPR-Cas gene editing and derivative technologies like base editors and prime editing allow precise correction of pathogenic mutations [20–22]. CRISPR-Cas9 systems derived from different bacterial species vary in their properties for gene targeting specificity and efficiency [23–28]. The most popular variants were discovered in *Streptococcus pyogenes* (SpCas9) and *Staphylococcus aureus* (SaCas9), the latter being smaller and thus preferred for AAV delivery [27, 29, 30]. Preclinical studies have demonstrated long-term efficacy of CRISPR-Cas based in vivo gene therapies in mice and even in larger animals and non-human primate models in various indications from hematologic diseases to metabolic and muscle disorders [31–37]. Many of these approaches rely on traditional gene therapy vectors, including AAV. Based on the successful preclinical study by Maeder et al. [37], the first CRISPR-Cas9 in vivo gene therapy, which began patient enrollment in the first half of 2020, utilizes an AAV delivered by sub-retinal injection into the eye to fix Leber congenital amaurosis, a severe form of retinal dystrophy. Here, delivery of a SaCas9 nuclease pair is used to excise an aberrant splice donor in intron 26 of the CEP290 gene, thereby eliminating the cryptic exon that leads to a premature stop codon and bi-allelic loss of function in the retina (ClinicalTrials.gov Identifier: NCT03872479). Recently, a second landmark clinical trial has been initiated, in which lipid nanoparticles containing SpCas9 mRNA and single guide (sg)RNAs are delivered systemically to target the liver of patients suffering from hereditary and advanced stage transthyretin amyloidosis (ClinicalTrials.gov Identifier: NCT04601051). To reduce the liver’s production of transthyretin and avoid further complications of excessive transthyretin-deposits in other organs, the investigators aim to disrupt the transthyretin gene in the patients’ livers as demonstrated in preclinical work in mice and rats [36].

### Early evidence for Cas9 immunogenicity in mice

**Fig. 2:**
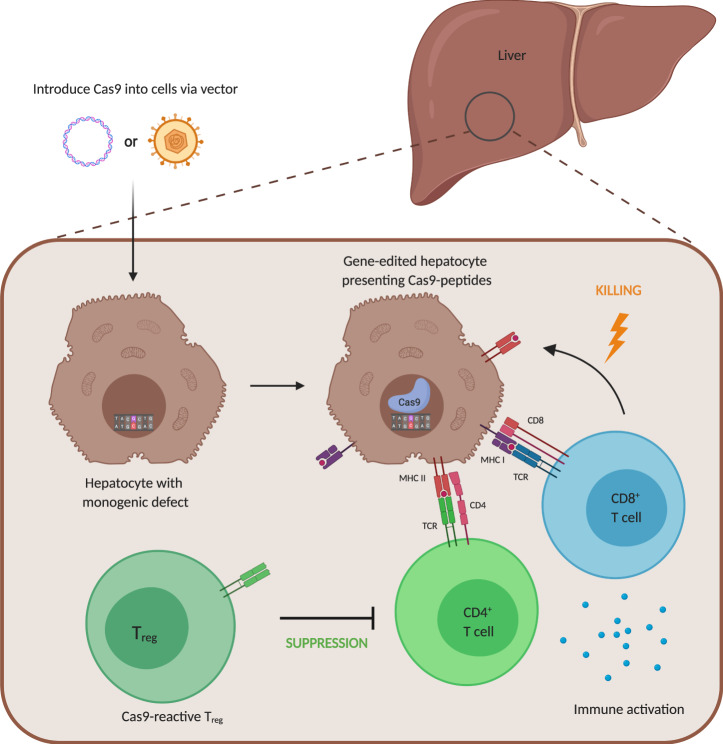
Immunological risk for anti-Cas9 T cell-mediated rejection of gene edited cells/tissues. CRISPR-Cas9 in vivo gene therapy requires Cas9 expression. Intracellular protein degradation processes lead to peptide presentation of Cas9 fragments on the cellular surface of gene-edited cells that may be recognized by SpCas9-reactive T cells. An excessive effector T cell (Teff) response could impact success of gene therapy. However, in vitro studies demonstrate anti-SpCas9 CD4^+^ Treg cells could inhibit the proliferation and function of anti-Cas9 Teff cells at high Treg to Teff ratios. Potential solution: Enhancing anti-Cas9 Treg cell responses prior to or during in vivo CRISPR-Cas9 gene-therapy.

Despite numerous successes in preclinical studies, SpCas9 nuclease proteins have been found to elicit both antibody and T cell responses in immunocompetent mice when delivered via adenovirus to livers [38] or when overexpressed as a transgene in muscle using AAV vectors or electroporation [38–40]. SpCas9-overexpressing tumors transplanted into immunocompetent mice are rejected by a Cas9-directed T cell response [41]. Importantly, a recent paper by Li et al. demonstrated that immunization of mice with SaCas9 protein one week prior to AAV-liver gene therapy prevented long-term survival of gene edited hepatocytes in vivo [42] (Fig. 2). It remains to be elucidated whether classical immunosuppressants, used clinically and in large animal models, could mitigate such T cell memory responses after AAV administration [2, 33].

## Human adults display preexisting adaptive Cas9-immunity

The most popular Cas9 nucleases are derived from facultative pathogenic bacteria suggesting that most humans are repetitively exposed to these strains due to infections or colonization [27]. Multiple teams—including our group—reasoned that such exposure could lead to adaptive immune memory toward Cas9, which led to the recent characterization of preexisting immunity to SpCas9 and SaCas9 in adult humans [43–45]. Results regarding SpCas9-specific antibodies are still debated due to high variability between the findings of Charlesworth et al. (58%), Ferdosi et al. (≥8.8%), and Simhadri et al. (5%) [43, 45, 46]. With respect to preexisting T cell immunity toward SpCas9 and SaCas9, the consensus is that the majority of healthy adults exhibit IFN-γ secreting T cells that are readily detected in the peripheral blood using different assays, such as flow cytometric analysis of activation markers and cytokines or IFN-γ ELISPOT (Summarized in Table 1) [43, 44, 46]. As expected for bacterial antigens, most SpCas9-reactive T cells belong to the CD4^+^ T cell compartment, nevertheless, initial studies also detected SpCas9-induced CD8^+^ T cell activation. Similarly, SaCas9 protein elicited T cell responses in the majority of tested individuals [43, 44]. In six donors, we detected a comparable T cell response after exposure to *Acidaminococcus species*-derived Cas12a (previously known as AsCpf1) [44]. Importantly, enriched SpCas9-reactive Teff cells were found to lyse SpCas9-overexpressing autologous B cells in vitro [44]. Therefore, human Cas9-reactive T cells have the capacity to recognize and eliminate Cas9-expressing cells in vivo. Traditionally, elimination of transgene-expressing cells is attributed to CD8^+^ T cells [2, 47], but a subpopulation of CD4^+^ T cells are also able to execute cytotoxic functions [48, 49].

**Table 1:** Summary of Cas9 directed T cell responses in humans.

Publication	SpCas9-sensitized donors	SaCas9-sensitized donors	AsCpf1-sensitized donors	Notes—agent for stimulation, analysis platform
Charlesworth et al. [43]	68% (*n* = 18)	78% (*n* = 18)	n/a	Whole recombinant protein, ELISPOT, flow cytometry
Wagner et al. [44]	95% (*n* = 48)	100% (*n* = 6)	100% (*n* = 6)	Whole recombinant protein, flow cytometry
Ferdosi et al. [46]	83%/90% (*n* = 12/*n* = 10 HLA-A2^+^)	n/a	n/a	In silico predicted peptide pools & HLA-A2 + pentamers, ELISPOT and flow cytometry
Stadtmauer et al. [57]	66.7% (*n* = 3)	n/a	n/a	Peptide pools, flow cytometry
Payne et al. [106] (WO 2017/081288 Al)	56–67% (*n* = 9)	n/a	n/a	Patent by LONZA LTD, Switzerland Whole recombinant protein, stimulation index in DC:CD4/CD8 assay

Most in vivo gene therapy targets are parenchymal cells within solid tissues with an important biological function (hepatocytes, retinal cells, muscle fibers), which express MHC class I under normal circumstances. A direct interaction between CD4^+^ T cells and gene-modified cells is unlikely, although MHC class II expression has been reported on a subset of parenchymal cells under inflammatory conditions [50–52]. Further, CD4^+^ T cells with T cell receptors (TCRs) restricted to MHC class I have also been reported [48]. Whether, CD4^+^ T cells contribute directly to damage to tissues modified with gene therapies is unclear. In-depth characterization of immunity toward Cas9 is therefore important to direct clinical treatment decisions for the safe application of CRISPR-Cas9 mediated gene targeting in vivo.

### Assessing the entire preexisting SpCas9-specific T cell repertoire

Monitoring of Cas9-directed immunity prior to in vivo gene therapy should aim to identify patients with a high frequency of preexisting pro-inflammatory T cell memory. Initial studies used full recombinant proteins to stimulate Cas9-specific T cells [43, 44], thus, the results are likely to favor CD4^+^ T cell responses, as classical antigen processing of whole proteins involves endocytosis and antigen-loading onto MHC class II molecules [53, 54]. However, cross-presentation by professional antigen-presenting cells results in SpCas9 antigen uptake, processing, and presentation on MHC class I [55]. Therefore, in the study by Charlesworth et al. and in our study, Cas9-activated CD8^+^ T cells were detectable. As an alternative to whole proteins, antigen-specific T cells can be efficiently stimulated with synthesized peptide libraries (pools) comprising oligopeptides spanning a given antigen of interest [56]. Recently, Ferdosi et al. reported 83% of 12 human donors displayed an IFN-γ^+^ T cell response after stimulation with a pool of 38 in silico predicted peptides for optimal MHC class I binding to the most prevalent haplotype in humans: HLA-A2:01. To allow for unbiased detection of the total Cas9-specific T cell repertoire, SpCas9-spanning peptide pools were generated by JPT Peptide Technologies. These peptide pools were recently used to detect T cell responses in cryopreserved leukapheresis products from patients in the first published results of CRISPR-Cas9 multiplex-gene edited and transgenic TCR-redirected T cells for cancer therapy [57]. Further, this peptide library lays the foundation for unsupervised epitope mapping to delineate a putative distinct epitope recognition of SpCas9 induced Teff and Treg cell responses [56]. A potential disadvantage of peptide libraries, however, are the limited peptide sizes, which could underestimate the number of T cells with specificity to longer, unconventional peptides, e.g., those which have been described as a target of CD8^+^ Treg [58].

Determining prior T cell sensitization through the activation marker CD137 indicates a very high to ubiquitous prevalence of SpCas9-reactive T cells [43, 44]. According to our results, a significant fraction of the SpCas9 activated CD4^+^ T cells display a phenotype associated with Treg cells [44]. As commensal bacteria are known to promote Treg cells at immunological surfaces, colonization with Cas9-expressing bacterial strains could have induced Cas9-specific Treg cells in humans.

### Cas9-reactive Treg cells in humans

Due to immunoregulatory functions of Treg cells, promoting endogenous cargo-specific Treg cells as well as transfer of cargo-specific Treg cells could serve as potent treatment alternative to improve the long-term outcome of in vivo gene therapy, without the need for extended conventional immunosuppressive treatments [10]. Enriched human SpCas9-reactive Treg cells can reduce proliferation as well as cytokine production of Cas9-reactive Teff cells during in vitro assays with a high Treg to Teff ratio [44]. Therefore, immune monitoring of both Cas9-directed effector and Treg responses in CRISPR-Cas clinical trials could provide information on whether a gene-therapy directed immune response is tolerogenic/suppressive or hazardous based on Teff/Treg ratio in peripheral blood. A misbalanced Teff/Treg ratio skewed toward high frequency of inflammatory cells or pronounced CD8^+^ T cell responses may allow risk stratification for future trials and potentially exclude high-risk patients for Cas9-associated in vivo gene therapy (Fig. 2). Similarly, Teff/Treg ratios have been used in other clinical indications to predict clinical outcomes, e.g., after allogeneic stem cell transplantation [59].

## Need for animal models that account for mixed preexisting immunity to Cas9

As classical animal models for gene therapy—including nonhuman primates—do not host human commensal bacteria like *S. pyogenes*, they do not reflect human immune sensitization. Thus, they are suboptimal for evaluating the impact of immunity on CRISPR-Cas in vivo treatments. As a first step towards a more complex model, Li et al. generated a mouse model with preexisting Teff cells by subcutaneous vaccination with small amounts of SaCas9 protein. One week after immunization, a liver-targeting AAV was systemically delivered. While editing and AAV-mediated GFP expression was initially observed in hepatocytes, after 12 weeks gene edited cells were eliminated, presumably by a CD8^+^ T cell response. This correlated with liver damage, indicated by histological examination and elevated liver enzymes in the serum. Interestingly, Li et al. detected an increase in the absolute frequency of Treg cells only after SaCas9 protein immunization, and therefore the increase in Treg numbers is unlikely to be related to AAV or other antigens. It was not investigated whether these Tregs are specific to SaCas9, which could, to a certain extent, explain their failure to prevent rejection of gene edited cells. Further characterization of this model is required to determine the extent to which a mixed preexisting immunity, consisting of both effector T cells and Tregs with specificity to Cas9, is present. In general, more models are required with preexisting immunity to Cas9. This might be of particular importance when investigating improved/tolerogenic vectors or co-treatments, because the ratio between antigen-specific Teff/Treg has been shown to predict the outcome of immune rejection (high: inflammation, low: tolerance).

## No immunogenicity events reported in immunocompromised patients receiving ex vivo cultured and SpCas9 edited cell products

The immunogenicity risk of CRISPR-Cas9 gene editing in humans largely depends on how and where the system is delivered. While in vivo Cas9 therapeutics add unique challenges to vector immunogenicity and host factors (discussed below), Cas9 delivery to in vitro cultured cells allows for more control over residual remnants within the final product. Ex vivo gene editing of cellular products with transient delivery methods (plasmid DNA, mRNA, protein) is expected to be safe in most cases as Cas9 is rapidly degraded and diluted in highly proliferating cells [60]. Early reports from clinical trials have already demonstrated persistence of SpCas9-edited T cell products and hematopoietic stem cells in human patients [57, 61, 62]. Stadtmauer et al. demonstrated that after electroporation of CRISPR-Cas complexes only minimal residual amounts of SpCas9 protein were detectable in the multiplex-edited T cell products prior to infusion [57]. Two of the three leukapheresis products that served as the source material displayed Cas9-specific T cell immunity [57]. Xu et al. and Lu et al. used DNA plasmids for CRISPR-Cas delivery to their respective cell products and did not report significant treatment-related adverse effects after infusion [61, 62]. Unfortunately, Cas9-directed immune responses were not measured in patients and cell products were not evaluated for remnants of Cas9 protein [61, 62]. As plasmids lead to high and longer expression than mRNA and protein platforms, this would have been particularly interesting in comparison to Stadtmauer et al. [63, 64]. None of the three studies compared SpCas9-specific immune responses before and after cell product application. All patients suffered from advanced malignant diseases and received either lymphodepletion or chemotherapy prior to cell infusion.

These results are encouraging regarding safety, although still very few patients were infused with CRISPR-Cas edited therapeutics and none of the trials were able to demonstrate clinical efficacy at this point. Additionally, all patients had compromised immune systems due to underlying disease and the treatment regimen prior to cell administration. Ongoing and future studies with ex vivo modified cell products should investigate humoral and cellular immune responses, because these phenomena could contribute to suboptimal treatment outcome by partial elimination of infused cell products.

## Immunogenicity issues of in vivo CRISPR-Cas therapeutics

In general, effective in vivo gene therapies must overcome three major immunological challenges: (1) delivery to the target cells without being neutralized by antibodies, (2) avoid detection and elimination by T cell memory responses toward the vector or cargo after delivery and (3) prevent the subsequent induction of immune responses towards the trans- or corrected healthy gene. Thus, CRISPR-Cas9 based strategies and preexisting Cas9-directed T cell immunity may add to the inherent immunogenicity problems of the viral vectors, which are discussed extensively elsewhere [2].

Most CRISPR-Cas therapeutics either aim to express Cas9 in target cells via nucleic acids or package the protein within a viral capsid or nanoparticle [65–68]. Therefore, anti-Cas9 pre-existing neutralizing antibodies should only alter success when Cas9 is exposed extracellularly and outside a vector hull, such as after direct injection into a tissue [69].

Cellular immunity to CRISPR-Cas is split into two components: (i) Innate immune control via danger or pathogen associated molecular patterns induces cytokines and chemoattractant substances for immune cells. This promotes and attracts the key mediators of (ii) the adaptive immune response including B cells and T cells. For example, nucleic acid sequences in the AAV vector genome trigger TLR9 and induce type I interferon [70]. In turn, interferons increase MHC expression on immune cells and even on the targeted epithelial cells [50, 70], thus initiating priming of the adaptive immune system and increasing the risk of Cas9 detection by preexisting memory T cells. Similarly, unmodified RNA ends of single guide RNAs induce interferons through RIG-1 signaling which impedes cell viability and reduces efficacy [71]. Removal of 5′-triphosphate ends or chemical modification of the sgRNA with 2′-O-methyl 3′phosphorothioate bonds reduces innate signaling and enhances genome editing in human cells [71, 72].

In contrast to conventional gene therapy, CRISPR-Cas gene editing allows long-lasting clinical effects with limited expression time. Regarding immunogenicity, “hit-and-run” approaches are particularly promising as T cell immunity is only effective as long as antigen fragments are displayed on the target cell’s surface. Optimized methods such as transient component delivery with self-inactivating vectors [73–75] or nanoparticles [36, 76] could thereby significantly reduce the risk of hazardous immune responses whilst retaining clinical efficacy.

### Managing immunity to CRISPR-Cas therapeutics

Certain organs including the eye and the central nervous system are considered immune privileged, with very low rates of immunogenicity related events in general, although a subset of patients do experience immunogenicity related events [77–81]. Cas9 proteins can be engineered to remove epitopes recognized by circulating cytotoxic CD8^+^ T cells [46]. Alternatively, Cas systems isolated from bacteria to which humans have not been exposed may circumvent the problem of preexisting immunity [82].

To delay and prevent immune responses clinically, multiple immunosuppressive drug treatments have been explored in viral gene therapy vectors [1]. Naïve immune responses can be attenuated by steroid drug treatment after ocular gene therapy [2, 83]. In other indications including hemophilia, oral immunosuppression fails to prevent cellular immune responses against AAV capsid proteins in a subset of patients, thereby limiting long-term transgene expression. Therefore, more intensive conditioning/immunosuppressive treatments are under investigation including T cell-depleting regimens [2, 84, 85]. Alternatively, preventing the processing or presentation of transgenes or vector antigens during initial viral gene delivery could be envisioned [86].

In the event that a single in vivo gene therapy dose induces an insufficient or transient clinical benefit, a second administration may be required to optimize treatment outcome. It is therefore important to consider the immunological consequences of repeated dosing. Moreno et al. proposed that immune orthogonality between Cas9 homologs and AAV serotypes could be exploited for sequential application of Cas-based therapeutics [87]. In a comprehensive report, they demonstrate that the immune responses to AAV and Cas homologs differed significantly despite moderate similarity between the protein sequences at certain epitopes (SpCas9 → AsCpf1 = 38% protein sequence identity, SpCas9 → SaCas9 = 26% protein sequence identity) [44]. This allowed for serial rounds of redosing in mice if very divergent AAV serotypes were used [87]. Whether this concept translates to humans with a preexisting polyclonal immune response needs to be elucidated. Importantly, the peptide sequence of the two HLA-A2-restricted immunodominant epitopes identified by Ferdosi et al. [46] (HLA-A*02:01; SpCas9_240-248 and SpCas9_615-62) are present in Cas homologs from other bacteria and even unrelated bacterial proteins, although it is unclear if these fragments are presented in vivo [42].

## Exploring Cas9-specific Treg cells to facilitate safe and efficient gene editing in vivo

High antigen expression in the liver has been shown to exhaust Teff and induce Treg cells directed against vectors and transgenes [11, 88]. Co-delivery of transgenes to the liver has been shown to promote tolerance in many other organs by transgene specific Tregs [2, 13, 89]. They can specifically remove MHC complexes from antigen-presenting cells [90], but also exert antigen-driven bystander suppression through mechanisms summarized earlier (Fig. 1). Similar to the liver co-delivery approach, boosting the number and migration of Cas9-reactive Treg cells may also serve as a platform to induce tolerance regardless of the targeted tissue.

Knowledge of the antigenic structures that mount the distinct Treg cell immune responses to Cas9 could allow for the creation of novel immunotherapies to promote immune tolerance for in vivo approaches with long-term Cas9 expression. We have previously shown that anti-Cas9 Teff and Treg cells may recognize distinct fragments of Cas9 as indicated by minimal clonal overlap between their TCR repertoire [44]. Similarly, this has been shown for both aeroantigens in allergy and neoantigens in cancer tissue [9, 91]. These unique possibilities should be exploited by:

(i) Adoptive transfer of Cas9-specific Treg generated by polyclonal expansion strategies used clinically [92, 93] or, for improved purity, adapted protocols with prior antigen-specific enrichment before expansion. Alternatively, gene editing and transfer of Cas9-specific TCRs into (induced) Tregs could be envisioned [94–96]. It has been shown previously that polyclonal ex vivo expanded Treg cells can prevent adaptive T cell responses to gene therapy [17]. A large body of evidence suggests that antigen-specific Treg cells are more potent than polyclonal products in transplantation and autoimmunity, as well as in gene therapy [18, 94, 97, 98]. Antigen-specific Cas9-reactive Treg cells could be expanded in cell culture and co-administered with CRISPR-Cas therapeutics [97, 98].
(ii) Creating an engineered Cas variant with mutated epitopes to avoid recognition by pre-existing Teff but preserves epitopes that stimulate Treg cells may circumvent the need for global immunosuppression in patients with pronounced pre-existing T cell immune reactivity to SpCas9. Accordingly, Ferdosi et al. created “immunosilenced” SpCas9 variants through targeted mutation of the two immunodominant epitopes recognized by pre-existing CD8^+^ T cells in HLA-A2^+^ donors while preserving function and specificity ([46] and similar approach in patent WO 2017/081288 Al). However, mutating more than two epitopes within the Cas9 protein might be required to avoid all preexisting Teff immunity, and it is unknown if such a multiply mutated Cas9 protein would retain full activity and specificity.

A final consideration for the safety of Treg induction/transfer is that high frequencies of Cas9-specific Treg cells may blunt immune responses toward facultative pathogens like *S. pyogenes*. Thus, a particular safety risk after Cas9-specific Treg cell induction/infusion could be an enhanced risk of invasive infection. Nevertheless, most adults have protective antibodies directed against tegument proteins of the pathogens and T cell immunity against other antigens. Regular anti-microbial immunity should be retained, as Treg cells do not block or inhibit secretion of antibodies by plasma cells.

## Final summary and outlook

Gene therapy is currently entering “a modern era”, with rising numbers of clinical trials taking place and the expectation that commercially available products will arrive within this decade [80, 99]. The discovery of CRISPR-Cas9 has significantly amplified the general excitement about gene therapies [19, 100]. As CRISPR-Cas based therapeutics race to the clinics, it is important to remember the history of gene therapy and its prior shortcomings regarding immunogenicity issues [1]. The death of 18-year old Jesse Gelsinger due to an over immune response to a high dose of adenoviral gene therapy vector halted the entire field of gene therapy for at least a decade [101]. Historically, viral gene therapies have struggled with toxicities especially after systemic application of high vector doses, some related to immunogenicity [102, 103]. Recently, two deaths were reported within an AAV trial for X-linked myotubular myopathy which mirrored the symptoms of liver failure observed in Jesse Gelsinger (although full data are not yet available as a peer-reviewed manuscript) [104]. Therefore, the identification of preexisting immunity to Cas homologs is timely and important to guide the design of better and safer clinical trials.

Preexisting immunity to Cas9 is a safety concern for in vivo application of the technology, that is unaccounted for in common model organisms, creating an urgent need for better models to evaluate CRISPR-Cas9 in vivo therapeutics. As Cas9-specific Teff and Treg cells can be enriched from peripheral blood in humans [105], this allows for the evaluation of their effects on the success of gene therapies using advanced in vitro models. Clinically, stringent immune monitoring could provide information on the role of endogenous Cas9-reactive Treg cell repertoires during first in vivo gene therapy trials. Potentially, immunotherapies to boost the frequencies of Cas9-specific Treg cells prior and during gene therapy may represent an attractive platform to promote tolerance. Therefore, CRISPR-Cas therapeutics could become a model in which to study the potential “beneficial alliance” between gene therapy and antigen-specific Treg cells [10].
